# Does the use of outdoor fitness equipment by older adults qualify as moderate to vigorous physical activity?

**DOI:** 10.1371/journal.pone.0196507

**Published:** 2018-04-30

**Authors:** Hsueh-wen Chow, Chia-Hua Ho

**Affiliations:** Graduate Institute of Physical Education, Health and Leisure Studies, National Cheng Kung University, Tainan, Taiwan; University of Rome, ITALY

## Abstract

Despite the rapid worldwide expansion of parks with outdoor fitness equipment (OFE), no objective data regarding the intensity of activity associated with using OFE are available. Hence, this study quantified the energy expenditure and intensity of physical activity by examining four outdoor fitness devices widely used by older adults and provides objective evidence-based intensity references for the Compendium of Physical Activities. Sixteen older adults (mean age: 70.7 ± 5.6 yr) equipped with a portable metabolic system for measuring energy expenditure and activity intensity completed tasks while walking or using four types of OFE. Descriptive statistics and repeated-measures ANOVA with the Bonferroni post hoc test were employed. The energy expenditure and activity intensity for using an air walker at tempos of 80, 100, and 120 bpm were 50.78 ± 14.76 (2.81 ± 0.85), 59.62 ± 14.23 (3.26 ± 0.82), and 65.62 ± 18.27 (3.55 ± 1.02) cal/kg/min (METs), respectively. The induced energy and intensity output values for a ski machine were 54.00 ± 14.31 (3.02 ± 0.87), 68.87 ± 22.74 (3.82 ± 1.35), and 74.55 ± 23.39 (4.05 ± 1.35) cal/kg/min (METs), at 80, 100, and 120 bpm, respectively. The energy output for a waist twister at 60 bpm was 38.43 ± 20.16 cal/kg/min (2.05 ± 1.15 METs), and that for a double arm stretch at 80 bpm was 31.05 ± 12.58 cal/kg/min (1.63 ± 0.70 METs). These findings indicate that activity on the ski machine and air walker could be considered to have moderate intensity, whereas the intensity of activity on the waist twister and double arm stretch was significantly lower than that for walking at either 3.2 km/h or 4 km/h and could be considered only light intensity. The MET values for the OFE were lower than those for similar indoor fitness equipment. The results of this study provide crucial implications for public health practices concerning the development of active living environments.

## 1. Introduction

Regular physical activity (PA) can counteract health problems [1, 2] such as chronic diseases [3], diabetes [4], cancer [5–7], and depression [8]; however, promoting regular participation in PA, which may mitigate these conditions, remains a challenge for public health [9, 10]. Accordingly, a growing body of literature addresses the importance of upgrading open green spaces or built environments to promote PA [11–19]. These approaches of improving environment have the potential to reach a broader, more sustainable base of citizens [20, 21] and are also in line with the aim of an aging-friendly city, as proposed by the World Health Organization [22].

The use of outdoor fitness equipment (OFE), or outdoor gyms, or family fitness zones has become very popular worldwide in numerous green spaces and built environments [23–28]. These facilities are modified configurations of conventional indoor gym equipment and are constructed of stainless steel and without power requirements for outdoor use. Several organizations have stated that OFE provides the community with free access to fitness training [26, 29], which is especially beneficial to people in low-income areas [30]. OFE is especially appealing to seniors throughout Asia, Europe, and North America [25, 31]. Parks equipped with OFE have acquired the moniker “senior playgrounds” [24, 25]. OFE attracts an increasing number of visitors to parks and provides them encouragement to be active. Studies have determined that the installation of OFE attracted new visitors to parks [27, 30] and was cost effective. Cohen et al. estimated that on average, visitors’ PA increased at a cost of only 10.5 cents per metabolic equivalent (MET) increase [30].

Although earlier studies [30, 32] have claimed that moderate to vigorous PA intensity (MVPA) was achieved using OFE, their measurements of energy expended using OFE appear to be arbitrary and subjective. For example, the analysis by Cohen et al. [30] failed to account for the variety of OFE. Some equipment may require extensive exertion to operate, whereas others might require only limited exertion. Even when employing the same OFE, different users have varying tempos or operating modes.

The MET is defined as the ratio of the energy expended for a given activity to the energy expended for quiet resting. The MET value is widely used to estimate PA intensity in surveillance for research and in clinical settings. The latest Compendium of Physical Activities published in 2011 [33] lists a wide array of PAs and their corresponding MET values (821 codes for specific activities within 21 major headings). To date, despite the popularity of OFE, no studies have objectively investigated the energy expenditure (EE) and level of intensity during OFE use. An earlier study suggested that a general MET range (3–6) representing a general intensity of PA during OFE use might oversimplify intensity measurement.

Thus, the aim of this study was to quantify the EE and intensity of exercise by using four popular types of OFE intended for older adults to provide objective evidence-based intensity references for the Compendium of Physical Activities [33]. In addition, the intensity levels during OFE use were evaluated and compared with those of similar activities reported in the Compendium of Physical Activities. More specifically, this study intends to

1. identify EE and METs for older adults using air walkers, ski machines, waist twisters, and double arm stretches;
2. classify PA during use of the four types of OFE into light, moderate, or vigorous;
3. examine the differences in EE and METs between the four OFE types and walking; and
4. compare the METs for the four OFE types with those for similar indoor gym facilities listed in the compendium.

## 2. Materials and methods

### 2.1. Participants

Sixteen recruited adults (eight men, eight women) older than 65 years, voluntarily participated in this study. For inclusion, the participants had to be healthy, without limitations for exercise. After an explanation of the details of the experimental protocols, such as the test procedures, potential discomforts, study locations, and ethical conduct, including the participants’ right to withdraw at any time during the study, the participants signed written consent forms before beginning the study. People were excluded from the study if they had contraindications to exercise or were not physically capable of completing the activities. The National Cheng Kung University’s ethics review board approved all procedures (HREC 101–102).

### 2.2. Instruments

**Fig 1:**
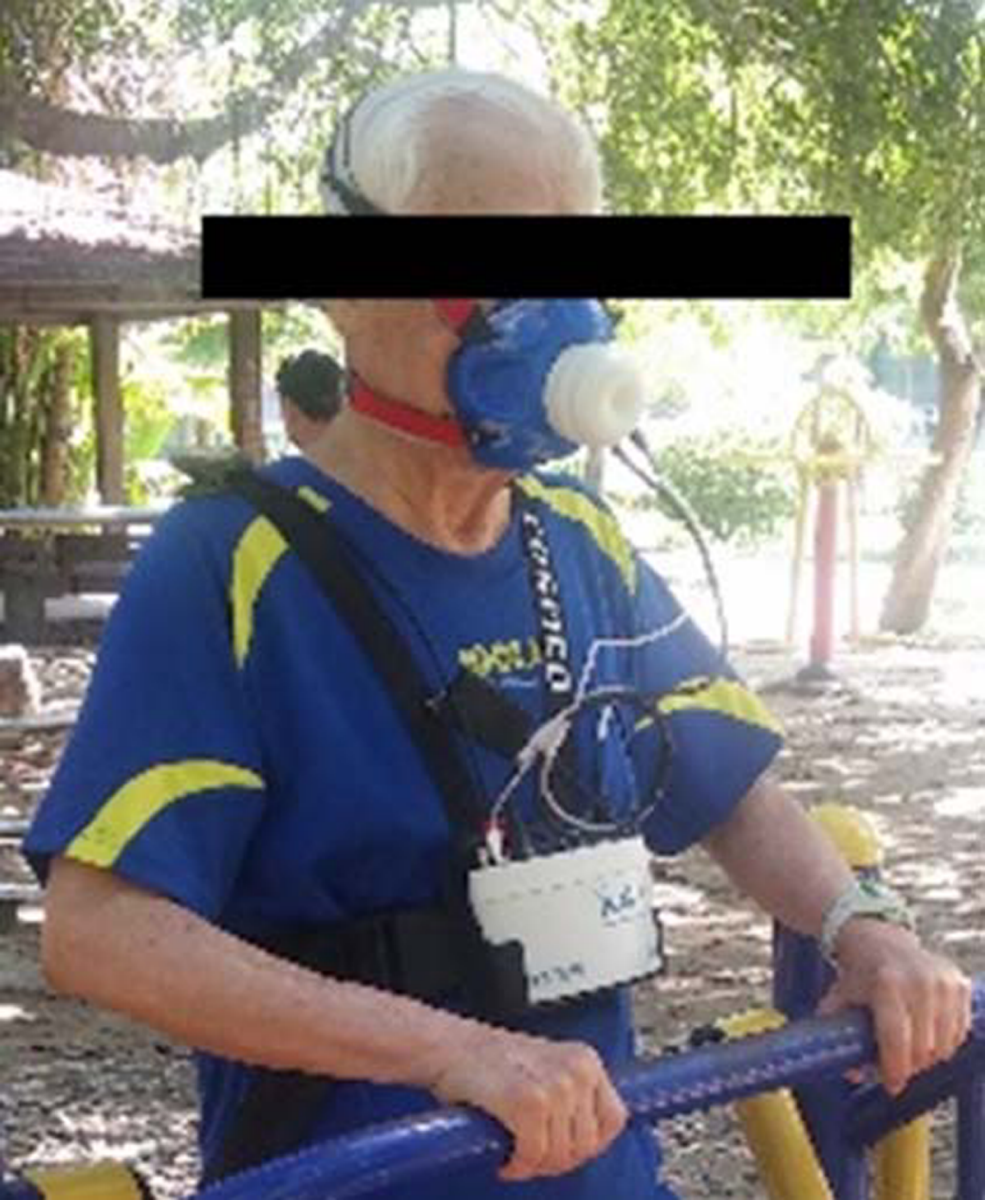
Study participant using an air walker in the park while equipped with the K4b2, with the mouth and nose tightly covered by a facemask.

Indirect calorimetric, direct calorimetric, and noncalorimetric measurements are commonly used for calculating the EE of humans. Each method has advantages and limitations in accuracy, reproducibility, reliability, and cost [34]. Respiratory indirect calorimetry is accurate, noninvasive, and highly reproducible and has been used in many studies [35]. To measure EE during an activity in non-laboratory field settings, the Cosmed K4b^2^ (Cosmed K4b^2^, Cosmed, Italy) was adopted in this study. The Cosmed K4b^2^ is a portable metabolic system that can measure breath-by-breath oxygen consumption (VO_2_) and carbon dioxide production (VCO_2_), which allows the quantification of the cardiorespiratory fitness of participants and prediction of EE during a specific activity. The system consisted of a portable unit and a battery weighing approximately 1.1 kg. The participants were required to wear this portable unit on their chest. They also wore a facemask that tightly covered their mouth and nose (see Fig 1). Prior to each individual test, the researchers followed the manufacturer’s guidelines (COSMED–SRL, 2010) for initializing and calibrating the system. A standard gas tank consisting of 16% carbon dioxide, 5% oxygen, and 79% nitrogen was employed. Participants also wore a polar belt to monitor their heart rate (Polar T34, UK).

### 2.3. Procedures

The experiment comprised four steps. Firstly, the participants completed a short survey questioning regarding the frequency of their visits to parks and use of OFE. Secondly, the resting metabolic rate (RMR) was obtained from the 1-minute steady state of a 3-minute measurement as a baseline for the EE of the participants. In accordance with the standard procedures for measuring RMR [36], the study participants were asked to refrain from food or beverage for 4 hours before the test and rest quietly for at least 30 minutes in the laboratory with the temperature controlled at 21°C–24°C. Thirdly, the participants walked at predetermined speeds of 3.2 and 4 km/h (2.0 and 2.5 mph, respectively) for 3 minutes on a treadmill in a laboratory setting to obtain a reference point [34] for the comparison of the experimental data with the data for other tasks. According to the Compendium of Physical Activities, the METs for walking at 2 and 2.5 mph are 2.8 (code: 17152) and 3 (code: 17170), respectively. PA intensities that are higher than those for walking are deemed higher than light-intensity PA. The walking speed was determined as studies indicated that older adults usually walk at a slow pace or steady average pace [37].

Finally, the participants moved to nearby parks equipped with OFE. The participants exercised on four OFE stations, namely the air walker, ski machine, waist twister, and double arm stretch, in a random order to control for the order effect. The four types of OFE were selected as they are the most popular among OFE users [38]. For the air walker and ski machine, the participants performed PA at three tempos, namely 80, 100, and 120 bpm. For the waist twister, the participants rotated their waists at 60 bpm. For the double arm stretch, the participants alternately pulled down the left and right handgrips at 80 bpm. These tempos were set on the basis of the average value and range of OFE users who operated this equipment in an earlier videotaped observational study [39]. A metronome was set at the desired beats per minute to help the participants maintain a regular tempo while performing each task.

**Fig 2:**
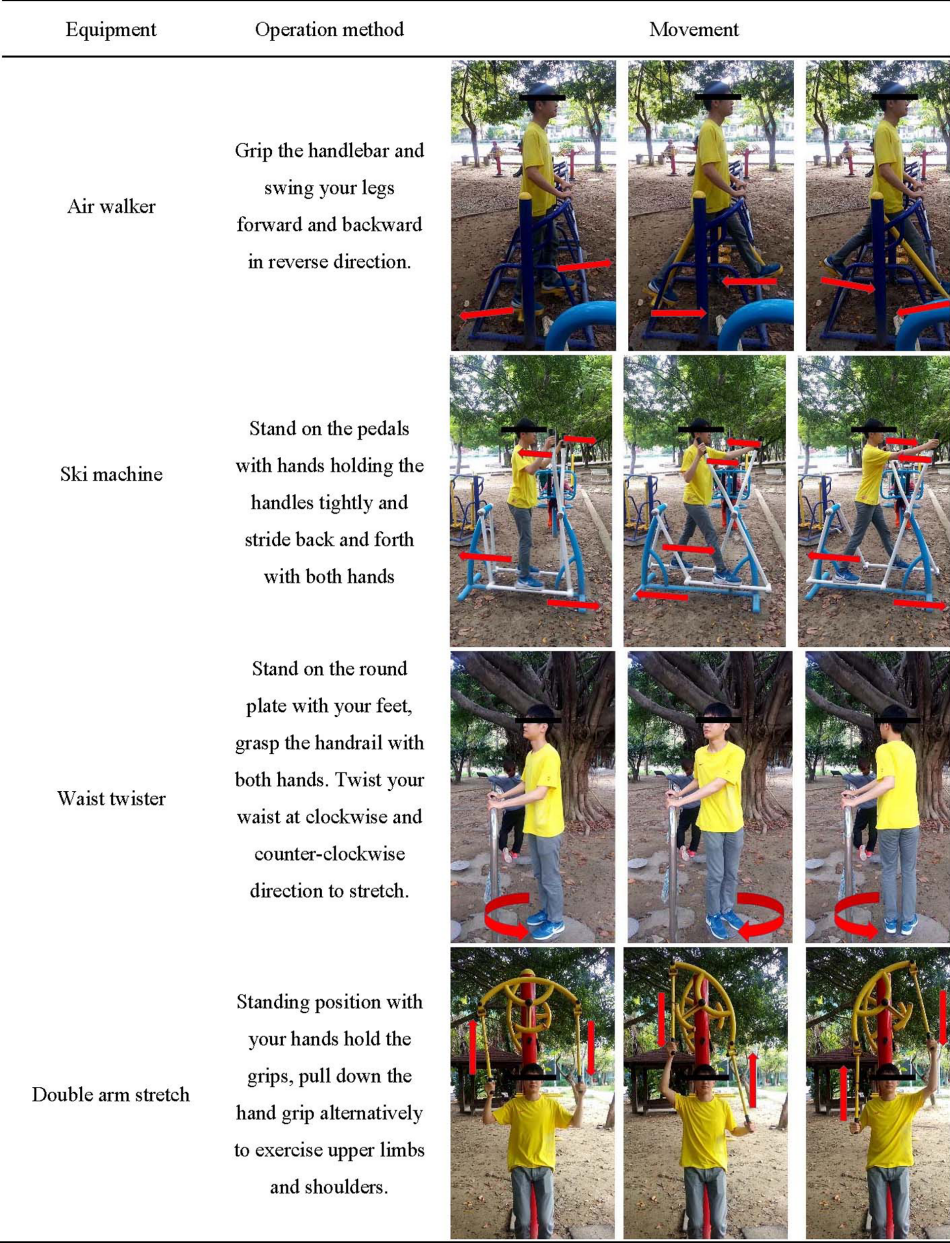
Four types of OFE in this study and the operation methods.

EE and METs during each task were measured. Each task was assessed for 3 minutes as referring to a previous experimental design which also used K4b^2^ to complete various exercise protocol [40]. The participants rested before performing another task to allow their heart rates to return to normal (as measured using the polar heart rate monitor). The participants received demonstrations for using each type of OFE before beginning each task, and their safety was assured by supervision. The operation method for each OFE is demonstrated in Fig 2.

### 2.4. Statistical analysis

The raw breath-by-breath pulmonary data from the Cosmed K4b^2^ software (version 9.1b), namely the VO_2_, VCO_2_, METs, energy expenditure per minute (EEm), and total energy expenditure (EEtotal), were examined and downloaded to a Microsoft Excel spreadsheet. Importing the data to the Matlab statistical software (Matlab 9.0, R2016a, USA) allowed the determination of the variations in EE and METs for each task. Because the numerous variations from the beginning to the end of each task might skew the data, previous studies have suggested selecting only steady-state values to reduce errors during periods of metabolic instability [41–43]. The results indicated that the data between 100 and 160 seconds achieved a steady-state stage. The average MET and EE data during this 60-second period represented the mean intensity and EE for performing each task for 1 minute. A repeated-measures analysis of variance (ANOVA) was performed to determine whether significant differences existed among the tasks. To meet the assumptions for repeated-measures ANOVA, normality was checked and Mauchly’s test of sphericity was used to measure the equivalent of the homogeneity of variance. In addition, partial η^2^ was used as a measure of effect size. Finally, when significant differences existed among the tasks, the Bonferroni test was used for post hoc comparisons [44, 45]. The level of significance was set at .05, and analyses were performed using the SPSS 17.0 software (IBM SPSS Inc.).

In addition, this study compared the intensity levels in terms of the MET values of the four OFE tasks with the values reported in the Compendium of Physical Activities for the corresponding PA categories to assess the differences in intensity between PA performed using OFE and similar activities [33].

## 3. Results

### 3.1. Participants’ background

The average age of the sixteen participants was 70.69 ± 5.59 years. Their average body mass index (BMI) was 23.6 ± 2.3 kg/m^2^. The mean RMR of the participants was 2.27 ± 1.20 mL/kg/min. No differences existed in the age, weight, BMI, and RMR; however, there were height differences between the men and women (Table 1).

**Table 1:** Descriptive statistics of the participants and differences between men and women determined using the t test.

		Group statistics			Levene’s test		t-test	
	Gender	*N*	*Mean*	*S*.*D*.	*F*	*sig*	*t*	*p*
Age	Male	8	69.8	6.5	0.70	0.42	-0.66	0.52
(yrs)	Female	8	71.6	4.7				
Height	Male	8	164.4	4.8	0.01	0.92	3.89	0.002**
(cm)	Female	8	155.0	4.9				
Weight	Male	8	63.4	10.6	3.98	0.07	1.24	0.24
(kg)	Female	8	57.8	7.3				
BMI	Male	8	23.3	2.8	4.00	0.07	-0.53	0.61
(Kg/m^2^)	Female	8	23.9	1.6				
RMR	Male	8	2.3	1.1	1.72	0.21	-0.03	0.98
(mL/Kg/min)	Female	8	2.3	1.3				

Note: ** p<.01

**Fig 3:**
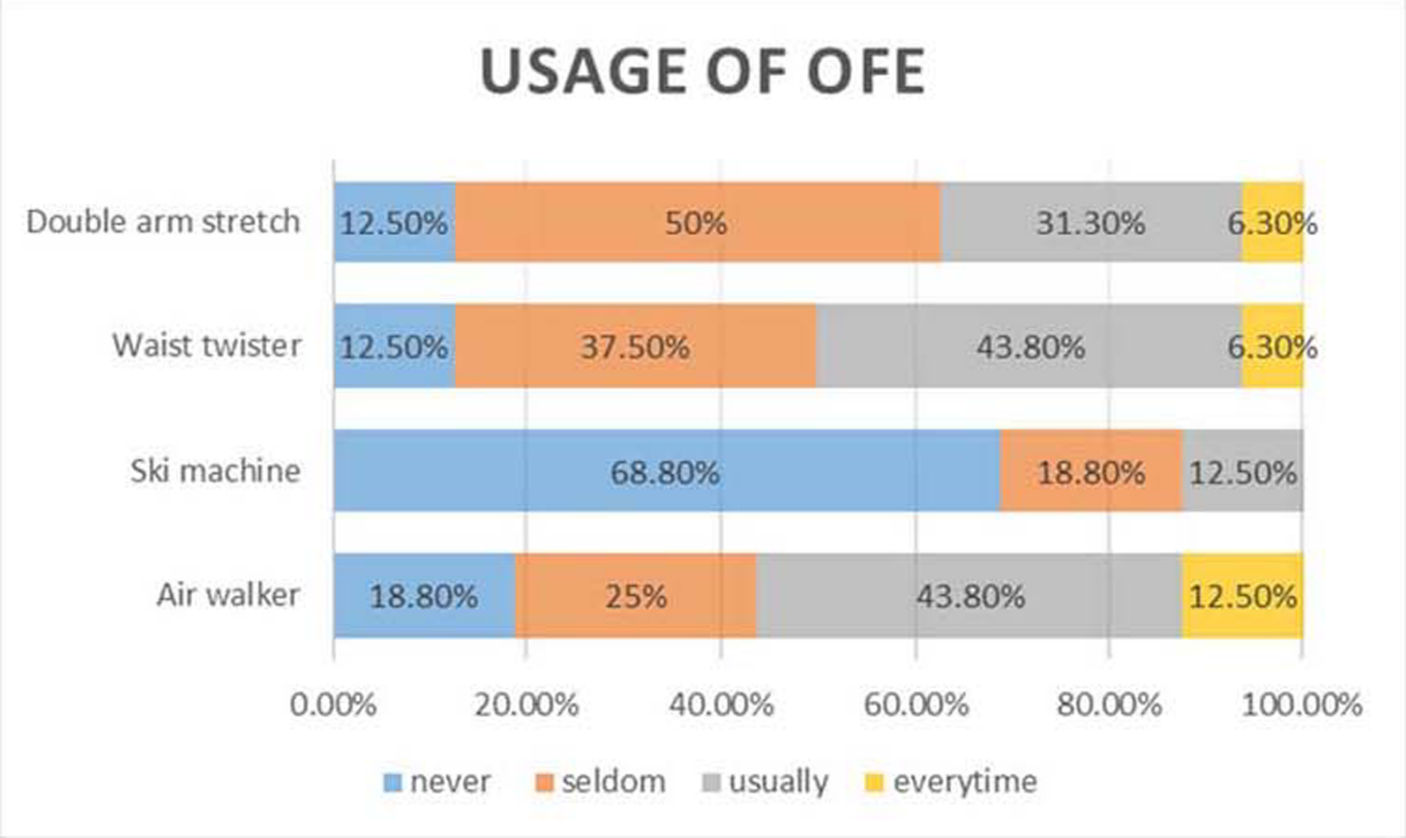
Frequency of OFE use at parks.

From the survey, 44% of the participants visited parks on a daily basis; 56% reported having habits for daily exercise; and 19% reported no regular PA. Regarding the frequency for using OFE at parks, the air walker and waist twister appeared to be popular among the participants, more than 50% of whom indicated that they used these machines frequently or every time they visited parks (Fig 3).

### 3.2. Energy expenditure and intensity during resting, walking, and activity on outdoor fitness equipment

Table 2 presents the EE and exercise intensity while the participants were resting, walking at 3.2 km/h or 4.0 km/h, and exercising on the four types of OFE. The data revealed that the average EE for the participants walking at 3.2 and 4.0 km/h were 58.53 ± 19.29 and 60.90 ± 20.69 cal/kg/min, respectively.

**Table 2:** EE and METs for each task and the differences among them determined using repeated- measures ANOVA and post hoc comparison.

Protocol	Total		Comparison^a^	
	Energy Expenditure cal/kg/min (mean ± S.D.)	Intensity METs (mean ± S.D.)	Sig. >	Sig. <
1.Rest	13.30±5.61	0.65±0.34		1<2,3,4,5,6,7,8,9.10.11
2.Walking (3.2 km/h)	58.53±19.29	3.23±1.04	2>1, 10,11	
3.Walking (4.0 km/h)	60.90±20.69	3.36±1.14	3>2,10,11	
4.Air walker (80 bpm)	50.78±14.76	2.81±0.85	4>1,11	4<9
5.Air walker (100 bpm)	59.62±14.23	3.26±0.82	5>1,10,11	
6.Air walker (120 bpm)	65.62±18.27	3.55±1.02	6>1,10,11	
7.Ski machine (80 bpm)	54.00±14.31	3.02±0.87	7>1,10,11	7<9
8.Ski machine (100 bpm)	68.87±22.74	3.82±1.35	8>1,10,11	
9.Ski machine (120 bpm)	74.55±23.39	4.05±1.35	9>1,4,7,10,11	
10.Waist twister	38.43±20.16	2.05±1.15	10>1	10<2,3,5,6,7,8,9
11.Double arm stretch	31.05±12.58	1.63±0.70	11>1,	11<2,3,4,5,6,7,8,9

Note: ^a^Post hoc paired comparisons by using the Bonferroni test. Significant differences at p < .05.

Table 2 reveals the mean and standard deviation of the EE for the participants’ activity on the air walker at 80, 100, and 120 bpm. The average EE for PA on the ski machine was marginally higher than that for PA on the air walker at the same tempo and order. The EE of the older adults when they used the waist twister station and double arm stretch station was much lower.

### 3.3. Intensity of activity (METs) during resting, walking, and exercise on outdoor fitness equipment

The intensity of activity was recorded in terms of the MET values for the examined tasks. A low MET value of 0.65 was recorded for resting. MET values of 3.23 and 3.36 were recorded for the two walking speeds at 3.2 km/h and 4.0 km/h. The MET values for performing PA on the air walker at various tempos (80, 100, and 120 bpm) ranged from 2.81 to 3.55. Performing PA on the ski machine required a high intensity, ranging from 3.02 to 4.05 METs. In accordance with the EE data, operating the waist twister and arm stretch required low effort levels; the MET values measured for each task were 2.05 and 1.63, respectively (see Table 2).

### 3.4. Comparison of intensity of activity (METs) for resting, walking, and exercise on outdoor fitness equipment

Table 2 also displays the results of the one-way repeated-measures ANOVA. Mauchly’s test of sphericity was violated (X^2^(2) = 90.813, p = .003); therefore, based on the rules of thumb [44, 45], the Huynh–Feldt correction was used. Significant differences in PA intensity were observed among tasks, as represented by the MET value and EE (F (6.098, 91.470) = 46.353, p < .0005, η^2^ = .756). Paired comparisons by using the Bonferroni test revealed that the PA intensity with the waist twister and the double arm stretch was significantly lower than that for walking at either speed. The PA intensity was the highest during use of the ski machine at 120 bpm, being significantly higher than the PA intensity with either the air walker or the ski machine at 80 bpm. Operating the same machine with different tempos resulted in different PA intensities. By contrast, the PA intensity was the lowest with the double arm stretch, being significantly lower than that with the other tasks.

Overall, these results indicate that for older adults, using the air walker and the ski machine represents moderate-intensity PA, and using the waist twister and double arm stretch can be considered only light-intensity PA according to the classification of the Compendium of Physical Activities (sedentary: <1.5 METs, light PA: 1.6–2.9 METs, moderate PA: approximately 3–6 METs, vigorous PA: > 6 METs) [33, 46].

## 4. Discussion

The promotion of health-enhancing PA by using OFE is becoming increasingly popular worldwide, especially for older adults [28, 38, 47–49]. This is the first study to objectively examine the EE and intensity of activity during OFE use by older adults. Among the four types of OFE, air walker and ski machine required more energy and reasonably represent moderate PA. Operating the waist twister and arm stretch required less EE and likely represents light PA. High energy was necessary to operate the air walker and ski machine because movements required forceful contractions of the engaged muscles in the lower limbs rather than in the arms or waist, which is in line with an earlier study indicating that the amplitude of contractions in the lower-limb movement is higher than that in the upper-limb muscles [50]. The low EE and MET values during rest might be due to the change in the body composition or other factors (e.g., substrate oxidation change) with the aging process, which is generally supported by other studies [51].

The results of this study also compared with the intensities listed in the compendium code for similar activities[33] (Table 3) because many pieces of OFE were modified from indoor gym equipment or other types of activities. In the current study, the intensities of the air walker and ski machine at 80, 100, and 120 bpm were lower than those with an indoor elliptical device, as indicated by a MET value of 5 (code: 02048) for the elliptical, moderate effort. In general, comparing the outdoor ski machine with indoor ski machines (code: 02080) revealed large gaps in the MET values. A possible explanation for these large gaps might be that the air walker and ski machine in this study did not adjust to various resistance levels. The conventional machines in indoor gyms have metal weight plates for resistance to induce strong muscle contractions with various speeds. However, many pieces of OFE only simulate the shape of the indoor equipment and lack the electrical power or weight to allow for modification of the intensity. Thus, most OFE requires minimum effort for operation.

**Table 3:** Comparison of the exercise intensity in METs for OFE with that for similar activities from the code of the Compendium of Physical Activities (Ainsworth et al. [32]).

Equipment	Current study	Compendium of Physical Activity	
		METs	Code #, (Description)
Air walker	2.81 METs(80bpm)	5.0 METs	Code:02048
	3.26 METs (100bpm)		(Elliptical, moderate effort)
	3.55 METs (120 bpm)		
Ski machine	3.02 METs (80 bpm)	6.8 METs	Code:02080
			(ski machine, general)
	3.82 METs (100 bpm)		
	4.05 METs (120 bpm)		
Double arm stretching	1.63 METs (80bpm)	2.3 METs	Code:02101
			(stretching, mild)
Waist twister	2.05 METs (60bpm)	2.5 METs	Code:09071
			(stand, miscellaneous)

With regards to the arm stretch and waist twister, no identical activity or equipment exists for indoor venues to allow a comparison. The code 02010 for mild stretching was the basis of comparison for the arm stretch. Code 09071 was the basis of comparison for the waist twister. The MET values for operating the arm stretch and waist twister were marginally lower than the corresponding values in the aforementioned codes (see Table 3).

The American College of Sports Medicine and the American Heart Association have recommended that older adults perform MVPA for a minimum of 30 minutes on 5 or more days per week to obtain health benefits. As indicated in this study, senior citizens may not achieve the desired moderate-intensity target with certain types of OFE.

## 5. Conclusions

Earlier studies, which subjectively claimed that the use of OFE achieves MVPA [30, 32] or arbitrarily assigned MET values to OFE use [52], are insufficient as they lack rigorous scientific measurement. This is the first study that focused on determining EE and intensity when older adults used OFE. The results revealed that the air walker and ski machine required high EE and intensity, and thus could represent moderate PA. Using the arm stretch and waist twister required low energy and represented light-intensity PA. In general, the use of OFE appeared to be less intense compared with the use of conventional resistance training machines in indoor gyms. Manufacturers could design OFE with innovative functions such as adjustable resistance [53] or progressive adaptable elements to allow for various resistance levels or diverse ranges of motion for strong muscle stimulus.

The results from this study are subject to certain limitations. Because EE was estimated in this study by using data collected from a portable breath-by-breath metabolic system, differences in the environmental pressure and temperature among regions might have affected the results. Despite the extensive varieties of OFE available, only the four most popular types of OFE were selected for measuring the intensity of activity and associated EE. The results may not represent all types of OFE. Future studies should be conducted on other types of OFE on the basis of the protocol of this study. In addition, the same type of OFE from different manufacturers might have differences in size, shape, materials, or smoothness of operation. These differences may contribute to variations in the force necessary for operation. Furthermore, in the current study, the participants followed the standard operating protocol for the use of OFE at set tempos; however, from field observations, users’ behaviors varied considerably. For example, some people may use the air walker with both legs moving forward and backward alternatively, some may move both legs simultaneously, and some may move only one leg at a time. People also have different stride lengths [39]. All these factors could affect EE and intensity of activity.

Notwithstanding these limitations, this study contributes to the existing knowledge by providing objective measures of the intensity and EE when older adults use OFE. The present study contradicts the general assumptions of earlier studies that using OFE represents MVPA [30]. This study provides additional information regarding accurate MET values for specific types of OFE. OFE facilities are rapidly expanding worldwide to promote PA, and a wide range of equipment is available on the market. This is the first study reporting both the EE and intensity of activity (METs) of older adults using OFE. This information is fundamental, and researchers should use it with cautions to assess the PA level during OFE use. A practical recommendation for manufacturers and policy makers is to provide the intensity level or EE information for each type of OFE, similar to the nutrition labels on food products. Hence, users could easily make a quick and informed choice of equipment. The results of this study are essential for helping public health policy makers evaluate the effectiveness of OFE interventions in promoting active aging among older adults.

## Supporting information

S1 File Experiment data. This data file contains all experiment data. Including participants’ backgrounds (gender: male = 1, female = 2; height in cm, weight in kg.) and Energy expenditures and METs value for 11 tasks (Re = rest, W1 = walking 3.2 km/h, W2 = walking 4.0 km/h, A1-A3 = air walker at 80, 100, and 120 bpm, S1-S3 = ski machine at 80, 100, and 120 bpm, T = twister, U = double arm stretch).
(PDF)
