# Supplementary material for: Does the use of outdoor fitness equipment by older adults qualify as moderate to vigorous physical activity?
Source: PLoS One. 2018 Apr 30;13(4):e0196507. doi: 10.1371/journal.pone.0196507 (PMC5927431; doi:10.1371/journal.pone.0196507)
Supplement: S1 File — This data file contains all experiment data. Including participants’ backgrounds (gender: male = 1, female = 2; height in cm, weight in kg.) and Energy expenditures and METs value for 11 tasks (Re = rest, W1 = walking 3.2 km/h, W2 = walking 4.0 km/h, A1-A3 = air walker at 80, 100, and 120 bpm, S1-S3 = ski machine at 80, 100, and 120 bpm, T = twister, U = double arm stretch). (PDF) [file pone.0196507.s001.pdf]

	ID	Gender	years	height	weight	BMI	E_A1	MET_A1
1	1	2	68	162	68	25.91	68.02	3.93
2	2	2	75	157	60	24.34	38.38	2.09
3	3	2	66	159	65	25.71	22.49	1.22
4	4	1	65	171	73	24.96	50.99	2.82
5	5	2	66	158	62	24.84	35.84	2.05
6	6	2	79	148	50	22.83	37.60	2.09
7	7	1	66	160	50	19.53	65.19	3.41
8	8	1	82	164	59	21.94	39.84	2.09
9	9	2	75	154	52	21.93	42.22	2.31
10	10	1	66	168	77	27.28	57.81	3.11
11	11	1	78	158	55	22.03	75.56	4.34
12	12	2	73	149	48	21.62	55.03	3.06
13	13	1	67	169	69	24.16	53.72	3.08
14	14	1	69	160	52	20.31	69.71	3.92
15	15	1	65	165	72	26.45	40.80	2.22
16	16	2	71	153	57	24.35	59.28	3.24

	E_A2	MET_A2	E_A3	MET_A3	E_Re	MET_Re	E_S1
1	72.51	4.04	55.48	2.93	18.55	1.04	59.56
2	33.34	1.76	26.08	1.35	8.18	.39	42.62
3	40.46	2.19	45.16	2.41	8.11	.37	39.04
4	46.99	2.57	66.12	3.63	12.55	.61	48.92
5	59.18	3.40	67.83	3.89	7.29	.33	41.51
6	57.97	3.15	67.17	3.50	11.28	.50	54.74
7	79.48	4.17	101.23	5.42	19.09	.88	70.49
8	43.18	2.27	53.16	2.68	6.47	.15	31.54
9	51.64	2.75	54.25	2.84	7.78	.30	33.55
10	52.37	2.78	64.42	3.51	9.77	.44	53.31
11	77.33	4.38	84.81	4.63	11.30	.49	79.59
12	62.19	3.33	70.38	3.77	21.98	1.12	51.75
13	67.58	3.72	58.52	3.27	12.45	.66	51.79
14	80.49	4.49	79.67	4.49	21.60	1.25	71.00
15	64.71	3.66	92.37	5.01	14.27	.69	68.18
16	64.48	3.54	63.21	3.41	22.12	1.16	66.38

	MET_S1	E_S2	MET_S2	E_S3	MET_S3	E_T1
1	3.34	58.33	3.08	70.59	3.68	46.00
2	2.27	37.42	1.95	42.83	2.26	20.37
3	2.15	44.17	2.41	40.89	2.25	20.82
4	2.70	74.75	4.22	68.31	3.61	27.46
5	2.37	83.78	4.68	92.10	5.11	31.59
6	3.19	67.10	3.91	74.17	4.14	21.89
7	3.86	98.31	5.37	101.54	5.55	86.65
8	1.73	34.43	1.87	39.41	2.08	24.29
9	1.80	40.76	2.22	45.43	2.29	19.76
10	2.69	50.70	2.52	62.37	3.15	40.53
11	4.58	90.18	5.06	95.21	5.20	34.21
12	2.85	78.11	4.24	96.57	5.15	32.83
13	2.95	75.20	4.25	68.00	3.77	42.19
14	4.22	109.68	6.35	105.71	5.98	74.70
15	4.02	87.86	5.08	93.95	5.29	63.07
16	3.68	71.14	3.87	95.64	5.23	28.48

	MET_T1	E_U1	MET_U1	E_W1	MET_W1	E_W2	MET_W2
1	2.50	43.72	2.45	74.88	4.33	58.82	3.38
2	1.02	18.19	.87	31.48	1.72	35.58	1.94
3	1.11	13.10	.67	50.89	2.76	41.67	2.24
4	1.47	27.33	1.46	59.84	3.36	66.33	3.66
5	1.69	33.27	1.82	33.65	1.89	38.55	2.23
6	1.00	23.75	1.19	65.69	3.53	65.96	3.71
7	4.56	54.40	2.58	115.03	6.07	124.11	6.72
8	1.23	15.23	.72	50.92	2.77	51.63	2.78
9	.96	15.45	.73	48.36	2.49	48.37	2.50
10	2.21	19.90	.90	42.73	2.29	50.14	2.70
11	1.74	38.91	2.04	69.90	3.78	77.97	4.28
12	1.69	36.55	1.94	58.85	3.29	63.31	3.46
13	2.30	35.02	1.97	50.84	2.94	54.88	3.11
14	4.30	48.78	2.73	63.55	3.77	73.02	4.27
15	3.55	35.12	2.00	55.90	3.16	59.48	3.28
16	1.49	38.13	2.02	64.00	3.55	64.50	3.53
